# Complete mitochondrial genome and phylogenetic position of *Filchneria songi* in Perlodidae (Insecta: Plecoptera)

**DOI:** 10.1080/23802359.2021.1997660

**Published:** 2021-11-12

**Authors:** Xin-Tong Li, Zhi-Teng Chen

**Affiliations:** a College of Life Sciences, Hainan Normal University, Haikou, China; b School of Grain Science and Technology, Jiangsu University of Science and Technology, Zhenjiang, China

**Keywords:** Mitochondrial genome, Perlodidae, *Filchneria songi*, phylogeny

## Abstract

The complete mitochondrial genome of the perlodid stonefly, *Filchneria songi* Chen, 2019 was sequenced and analyzed. This double strand, circular molecule is 16,028 bp in length with an A + T content of 70.1%, and contains 13 PCGs, 22 tRNA genes, and two rRNA genes. A 1099-bp long control region was detected, with a high A + T content of 81.9%. Gene arrangement was conserved in the mitogenome of *F. songi*. Most PCGs use standard start codons and ended with complete stop codons. The phylogenetic analysis supported that *F. songi* was closely related with species of *Perlodes* Banks, 1903.

*Filchneria songi* Chen, 2019 (Plecoptera: Perlodidae) is an endemic stonefly known from Shaanxi Province of China. However, very few genetic information is available for *F. songi*, which is unfavorable for the resolution of phylogeny in Perlodidae. To provide more molecular data for *F. songi*, we sequenced and analyzed the complete mitochondrial genome (mitogenome) of this stonefly as the first representative for genus *Filchneria* Klapálek, 1908. A phylogenetic analysis was also performed to confirm its position in the family Perlodidae. The specimens of *F. songi* were collected from Taiping National Forest Park, Xi’an City, Shaanxi Province, China (33.9077 N, 108.6513 E, 1200 m) in April 2019. All specimens and DNA samples were stored in the Insect Collection of Jiangsu University of Science and Technology (ICJUST, Zhi-Teng Chen, 741208116@qq.com, under the voucher number ICJUST-PO1), Jiangsu Province, China. The sequencing strategy followed that of Chen and Du (2018), including an initial amplification of the large and small ribosomal genes (*rrnL* and *rrnS*), *cox1* and *cox2* genes, followed by LA-PCR amplifications which obtained two overlapping fragments covering the whole mitogenome. The PCR fragments were sequenced in Map Biotech Company (Shanghai, China). Structural and phylogenetic analyses also followed that of Chen and Du (2018). The mitogenome sequence of *F. songi* was deposited in GenBank with the accession number MZ475123.

The complete mitogenome of *F. songi* has a length of 16,028 bp and contains an A + T content of 70.1% (A: 36.5%, T: 33.6%, C: 18.0%, G: 11.9%). A typical set of 37 genes (13 PCGs, 22 tRNA genes, and two rRNA genes) and a long non-coding control region were annotated. The control region was located between *rrnS* and *trnIle*, 1099 bp in length with an A + T content of 81.9%. The gene rearrangement of *F. songi* is highly conserved among stoneflies.

Most PCGs started with the standard start codon ATN (ATT and ATG), whereas *ND1* initiated with TTG. Most PCGs terminated with the complete stop codon TAA or TAG, whereas *COX2* and *ND5* ended with an incomplete codon T. The 22 tRNA genes varied in length from 59 bp to 71 bp, mostly showing clover-leaf secondary structures except for *trnSer1 (AGN)* and *trnTyr*, which showed reduced tRNA arms. The two rRNA genes, *rrnL* and *rrnS* are found in the conserved locations as in other stoneflies. The *rrnL* gene was 1333 bp in length with an A + T content of 73.7%; the *rrnS* gene was 797 bp in length with an A + T content of 68.3%. There were 33 overlapped nucleotides between nine gene pairs with the longest overlap between *trnTrp* and *trnCys*. A total of 130 intergenic nucleotides were found between 13 gene pairs, with the longest intergenic sequence between *COX2* and *trnLeu2 (UUR)*.

Phylogenetic analyses for Perlodidae were performed using the concatenated nucleotide sequences of 13 PCGs. The BI and ML trees generated identical topologies (Figure 1). In both trees, *F. songi* sequenced in this study is supported as the sister group of *Perlodes* sp., another species from family Perlodidae. The monophyly of genus *Isoperla* Banks, 1906 was not supported by the mitogenomic data. The taxonomy of *Isoperla* was also considered controversial due to the loss of most holotypes (Chen et al. 2021). More molecular data including mitogenome data are required to solve the molecular phylogeny of Perlodidae.

**Figure 1.**
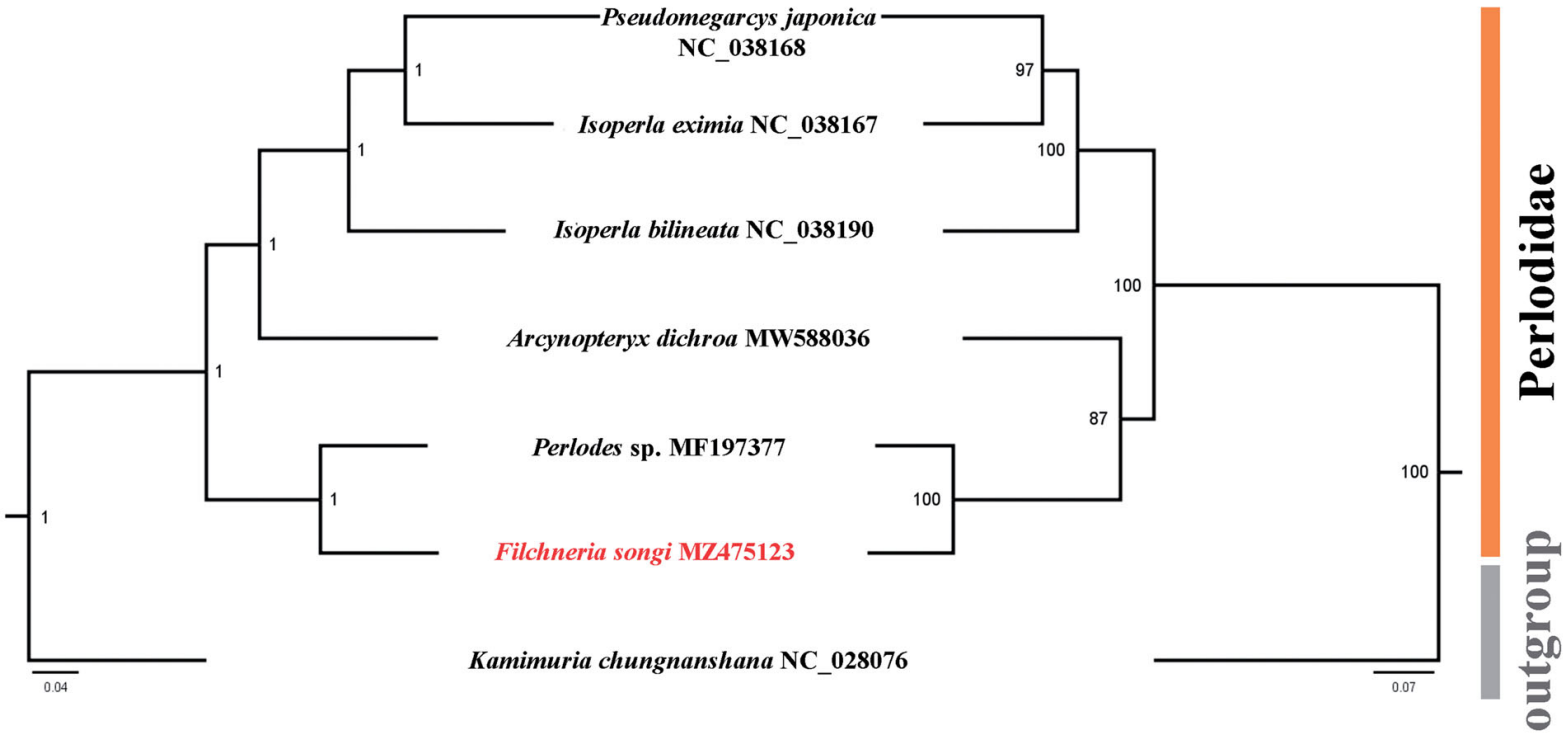
Phylogenetic tree of six species of Perlodidae. Numbers at the nodes are posterior probabilities of BI analysis (left value) and bootstrap values of ML analysis (right value). The GenBank accession numbers are indicated after the scientific names. The tree is rooted with *Kamimuria chungnanshana* (Perlidae, NC_028076).

## Data Availability

The genome sequence data that support the findings of this study are openly available in GenBank of NCBI at https://www.ncbi.nlm.nih.gov under the accession no. MZ475123.

## References

[CIT0001] Banks N. 1903. New name for *Dictyopteryx* Pictet. Ent News. 14:241.

[CIT0002] Banks N. 1906. On the perlid genus *Chloroperla*. Ent News. 17:174–175.

[CIT0003] Chen ZT. 2019. A new species and a new record of Perlodidae (Plecoptera) from China. Zootaxa. 4623(1):189–200. 10.11646/zootaxa.4623.1.13 31716283

[CIT0004] Chen ZT, Du YZ. 2018. The first two mitochondrial genomes from Taeniopterygidae (Insecta: Plecoptera): structural features and phylogenetic implications. Int J Biol Macromol. 111:70–76. 29292150 10.1016/j.ijbiomac.2017.12.150

[CIT0005] Chen ZT, Du SK, Li XT. 2021. Description of a remarkable new species of *Isoperla* (Plecoptera: Perlodidae), with supplements for *Isoperla kozlovi* Zhiltzova, 1972 from China. Zootaxa. 5027(2):160–174. 10.11646/zootaxa.5027.2.2 34811236

[CIT0006] Klapálek F. 1908. *Pteronarcys sachalina* sp. n., die zweite asiatische Art der Gattung. (Neuroptera, Plecoptera). Bull l’Acad Imperial Sci. 2(3):237–238.

